# Case of Vaginal Hysterotomy

**Published:** 1848-06

**Authors:** W. K. Scott

**Affiliations:** Buffalo


ART. V.—*Case of Vaginal Hysterotomy communicated by W. K. Scott M. D.*

About twenty-five years ago, I was called by Dr. Truman B. Hicks, of Warren County, in this State, to visit a patient of his, who had been in labour for more than a week, during which time no traces of the os tincae could be found. She was a healthy country woman who had borne children, and nothing peculiar appeared in the case except the absence of, or our inability to find, the mouth of the womb. The pains had been perfectly regular from the first; when exhausted, she would sleep quietly a short time, and on waking the pains would come on again.

When I first saw her, the pains were regular and strong. On examination I could discover no os tincae, but the uterus during the pains was pressed into the vagina, presenting an elastic tumour, through which, however, the head of the child could not be distinctly felt. On consultation, it was determined to make an opening into the uterus within the vagina, which

I did with a small scalpel guarded by the finger. It was done during a pain, and the opening was large. A gush of blood followed, and Dr. Hicks immediately proceeded to turn the child, which he accomplished in a short time, and safely delivered the patient. The placenta adhered to that portion of the uterus where the mouth is usually situated, and where the opening was made; this accounts for the amount of blood which escaped at first, and induced us to hasten the delivery.

The woman recovered as rapidly as usual, without any unpleasant symptoms, and I believe, has since, borne children, It was reported in the neighbourhood that during her pregnancy she had frequently endeavoured to introduce an iron instrument into the os uteri, for the purpose of producing an abortion. Of the truth of this report I know nothing; but at the time it struck me as the only means of accounting for the obliteration of the mouth of the uterus.

Should this be seen by Dr. Hicks, he will confer a favour upon the profession, by reporting further particulars of the case. As I never saw the patient but once, and that so long ago, I can only remember the most prominent facts.

Buffalo June 3, 1848

